# Morphological Study of the Thebesian Valve in Fresh Autopsied Adult Human Hearts

**DOI:** 10.7759/cureus.36534

**Published:** 2023-03-22

**Authors:** Shanmugam Shanthini, Hottigoudar Yekappa Suma

**Affiliations:** 1 Anatomy, Zoram Medical College, Falkawn, IND; 2 Anatomy, Jawaharlal Institute of Postgraduate Medical Education & Research, Puducherry, IND

**Keywords:** morphology, cannulation, coronary sinus ostium, human heart, thebesian valve

## Abstract

Background

The coronary sinus (CS) and its tributaries have been used to perform various electrophysiological and cardiac interventional procedures which require cannulation. The Thebesian valve (TV) guarding the coronary sinus orifice (CSO) exhibits morphological variations which might make cannulation unsuccessful leading to the failure of invasive cardiac procedures. This study aimed to analyze in detail the morphological features of the TV in fresh autopsied human hearts which were representative of the adult population of this region owing to its practical implications in invasive cardiac procedures.

Methodology

This was a cross-sectional, descriptive study conducted in the Department of Anatomy in collaboration with the Department of Forensic Medicine and Toxicology at Jawaharlal Institute of Postgraduate Medical Education and Research, Puducherry. A total of 104 fresh adult heart specimens were collected during the autopsy. The CSO was located, and the characteristic shape, composition, position, and extent of coverage of the CSO by the TV were observed and analyzed.

Results

The TV was present in 65% of heart specimens. The most common shape was remnant (33%), and the most common site of origin was inferior (63%). The valve composition was thin and membranous in 63% of heart specimens. In 7% of heart specimens, the TV covered more than 75% of the CSO diameter, of which in 4% of heart specimens, the CSO was completely closed and found to be obstructive.

Conclusions

This study highlights the variability in the morphological structure of the TV in adult human hearts and its potential implications in unsuccessful CS cannulation and failure of invasive cardiac procedures. Thus, prior imaging of the TV should be an integral part of CS cannulation procedures to avoid unsuccessful cannulation and complications related to repeated forceful cannulation.

## Introduction

The coronary sinus (CS) is the major venous trunk draining 60% of venous blood from the heart to the right atrium. Its orifice is situated in the right atrium posteromedially between the right atrioventricular orifice and the inferior vena cava. The coronary sinus orifice (CSO) is guarded by the Thebesian valve (TV), which is embryologically the caudal remnant of the right sinoatrial valve [1].

In this era of percutaneous transcatheter therapies for cardiac diseases, the cannulation of CS has become inevitable. The CSO, the primary point of entry into the coronary venous network, is guarded by the TV which shows a prevalence of 65-95%, along with a large variability in its morphology [2]. The CSO has been cannulated to perform various diagnostic and therapeutic invasive cardiac procedures such as cardiac resynchronization therapy, catheter ablation of cardiac arrhythmias, retrograde cardioplegia administration, targeted drug delivery, and percutaneous mitral annuloplasty [3,4]. Clinical data suggest that CS cannulation is unsuccessful in 5-10% of patients undergoing invasive cardiac procedures [5]. The causes for inadvertent cannulation have been ascribed to significant morphological variations of the TV in the form of its shape, size/height, position, composition, and extent of coverage of the CSO by the valve.

Data of morphological variations of the TV described in earlier studies were from formalin-fixed cadaveric hearts. The goal of this study was to highlight in detail the morphological features of the TV in fresh autopsied human hearts, which are lacking in the adult south Indian population, owing to its practical implications in invasive cardiac procedures.

## Materials and methods

This cross-sectional, descriptive study was conducted in the Research Laboratory of the Department of Anatomy in collaboration with the Department of Forensic Medicine and Toxicology at Jawaharlal Institute of Postgraduate Medical Education and Research, Puducherry from June 2015 to June 2017. This study was approved by Postgraduate Research Monitoring Committee and Institute Ethics Sub-committee (Human Studies) vide approval number JIP/IEC/SC/2015/19/781 dated July 17, 2015.

After obtaining written informed consent from the close relatives of the deceased, 104 fresh adult heart specimens of both sexes aged 21-86 years were obtained during the autopsy by convenient sampling technique method after being subjected to inclusion and exclusion criteria. Exclusion criteria included trauma, previous history of heart surgery, myocardial infarction, cardiomyopathy, and pericarditis. Baseline parameters of the deceased such as name, age, gender, residential address, hospital number, postmortem number with date, the weight of the heart, and the cause of death were noted in the data collection proforma.

**Figure 1:**
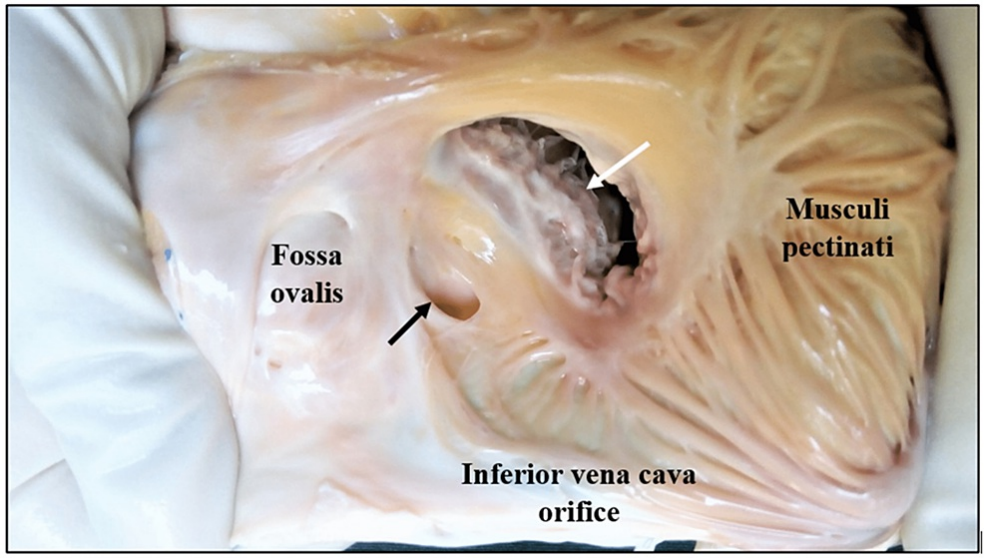
Interior of the right atrium showing the coronary sinus orifice without the Thebesian valve. Black arrow: the coronary sinus orifice; white arrow: the tricuspid orifice. Image credit: Shanthini Shanmugam

**Figure 2:**
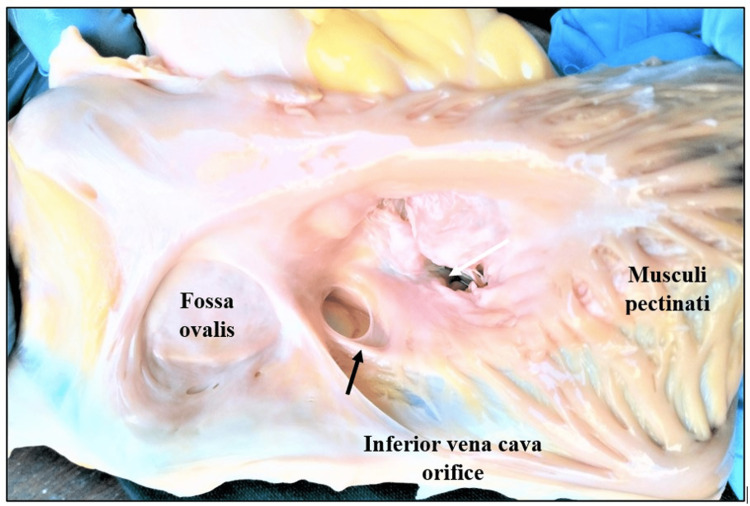
Interior of the right atrium showing the coronary sinus orifice with the Thebesian valve. Black arrow: the coronary sinus orifice; white arrow: the tricuspid orifice. Image credit: Shanthini Shanmugam

Heart specimens were washed thoroughly to remove blood stains, and the right atrium was opened by making an incision close to the sulcus terminalis along the right border of the heart from the superior vena cava to the inferior vena cava. The interior of the right atrium was exposed, the CSO was identified, and the presence of the TV and its morphological features were noted, documented, photographed in situ (Figures 1, 2), and characterized based on the Classification of Thebesian Valve, as recommended by Holda et al. [6]. The parameters studied included occurrence (the presence or absence of valve), shape, origin/position (the site of attachment in the CSO), nature/composition, and the extent of CS ostial coverage by the valve. Data were summarized as mean and corresponding standard deviations (SDs). All statistical analyses were done using SPSS Statistics version 19.0 for Windows (IBM Corp., Armonk, NY, USA).

## Results

A total of 104 fresh autopsied adult hearts were observed and interpreted. Out of the 104 heart specimens, 71 (68%) were males and 33 (32%) were females. The age ranged from 21 to 86 years, with a mean (SD) of 45 (16.2) years.

**Figure 3:**
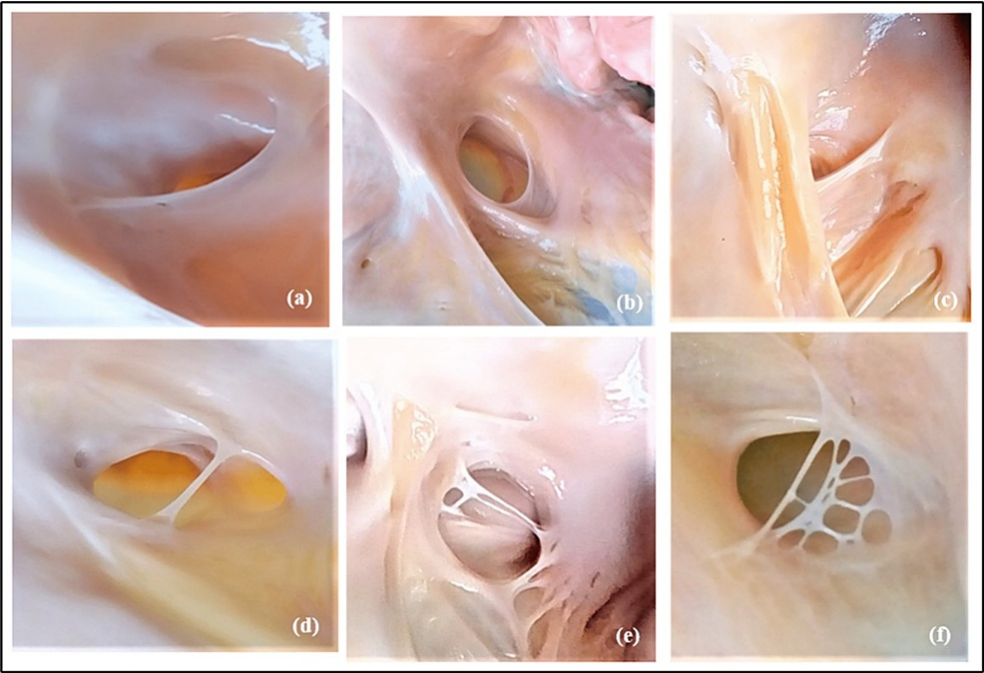
Morphological types of the Thebesian valve. (a) Remnant, (b) semilunar (c), fold (d), cord (e), mesh, and (f) fenestrated. Image credit: Shanthini Shanmugam

The CSO was present in all heart specimens in normal locations between the inferior vena cava and the tricuspid orifice. Then heart specimens were examined for the occurrence of the TV. The TV was present in 65% (68/104) and absent in 35% (36/104) of heart specimens. Thereafter, the shape of the valve was observed. Six types of valves, namely, remnant, semilunar, fold, cord, mesh (valve with multiple cords), and fenestrated (valve with multiple openings), were observed (Figure 3, Table 1).

**Table 1:** Morphological types of the Thebesian valve.

Shape of the Thebesian valve	Number of heart specimens with the valve (N = 68)	Percentage (%)
Remnant	22	33%
Semilunar	15	22%
Fold	9	13%
Cord/Band	7	10%
Fenestrated	6	9%
Mesh	9	13%

The most common type was remnant, followed by semilunar, which constituted 33% (22/68) and 22% (15/68), respectively. Two cases in fold type and one case in fenestrated type were completely closing the CSO which constituted 4% (3/68).

The inferior position of the TV was dominant with a frequency of 63% (43/68). Subsequent morphologies included the valve along the posterior position in 30% (20/68) and the anterior position in 7% (5/68), while the superior margin of the CSO was free from valve attachment (Table 2).

**Table 2:** Position of the TV in the CSO. CSO: coronary sinus orifice; TV: Thebesian valve

Position of the TV in the CSO	Number of heart specimens with the valve (N = 68)	Percentage (%)
Inferior	43	63%
Posterior	20	30%
Anterior	5	7%
Superior	-	-

The composition (nature) of the TV was assessed subjectively and observed to be thin and membranous in 63% (43/68), thick and fibrous in 24% (16/68), and mixed in 13% (9/68) of heart specimens (Table 3).

**Table 3:** Composition of the Thebesian valve.

Composition of the Thebesian valve	Number of heart specimens with the valve (N = 68)	Percentage (%)
Thin, membranous	43	63%
Thick, fibrous	16	24%
Mixed	9	13%

The extent of coverage of the CSO by the TV was assessed subjectively. In 35% (36/104) of heart specimens, the TV was absent and the CSO was fully open. The remaining 65% (68/104) of heart specimens showed valves covering the CSO to various extents (Table 4).

**Table 4:** Percentage coverage of the CSO by the TV. CSO: coronary sinus orifice; TV: Thebesian valve

Extent of coverage of the CSO by the TV	Number of heart specimens (N = 68)	Percentage (%)
100%	3	4%
76–99%	2	3%
51–75%	7	10%
26–50%	9	13%
<25%	47	70%

**Figure 4:**
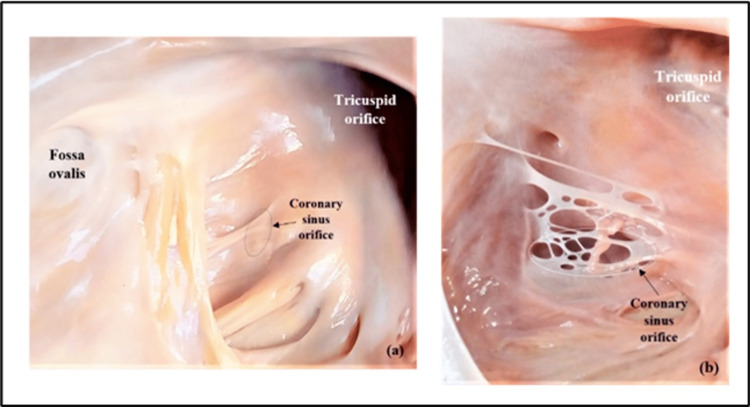
Obstructive type of the Thebesian valve. (a) Fold type and (b) fenestrated type. Image credit: Shanthini Shanmugam

In the majority of hearts (63/68), the extent of coverage of the CSO by the TV was <75%. In five out of 68 hearts studied, the valve covered >75% of the CSO, of which three valves were almost covering the CSO completely (Figure 4).

## Discussion

This study aimed to describe the morphological features of the TV in fresh autopsied human hearts which are representative of the adult population. The parameters studied included the morphology and position of the TV, composition of the valve, and extent of coverage of the CSO.

The TV exhibits variations in its occurrence, size, shape, and extent of coverage [6]. According to the literature, the occurrence of TV varies across different studies. Holda et al. [6] observed the TV in 82% of specimens, Ghosh et al. [7] in 79%, Randhawa et al. [8] in 64%, Mak et al. [5] in 73%, Hellerstein et al. [9] in 85%, Anh et al. [10] in 54%, and Christiaens et al. [11] in 36%. In this study, the TV was present in 65% of examined hearts, which is lower in comparison to the above cadaveric studies but higher than the imaging studies.

The shape of the TV is highly variable. Several authors have tried to classify the TV based on shape [5-8,10,12,13-17]. In this study, the classification suggested by Holda et al. was considered. Five types of TV have been described, with the most common type being type II (semilunar), which constitutes 32.6% [6]. In this study, all these five types were observed, with the most common being type I (remnant) present in 33%, followed equally by type II (semilunar) and type V (mesh and fenestrated), each constituting around 22%. The various shapes of TV have clinical significance during CS cannulation. Remnant and semilunar-shaped TV does not hinder CSO cannulation. While fold, cord/band, fenestrated, and mesh types which are membranous in nature and with ostial coverage <75% might cause difficulty in cannulation due to the possibility of entanglement of CS catheter, whereas if they were fibrous (unyielding) in nature and with ostial coverage >75%, they would obstruct CS cannulation leading to failure of interventional cardiac procedures.

The site of attachment of the TV was from the inferior margin (63%) or posterior margin (30%) of the CSO (Table 2). In five hearts (7%), the valve was attached at the anterior margin of the CS, close to the tricuspid orifice. No valves were found to originate from the superior margin of the CSO. This is in accordance with previous studies [6,7,10,12,16]. Thus, it can be suggested that for cannulation of the CS, an anterosuperior approach could be preferable.

The composition of TV was noted to be membranous in 63% (43/68), fibrous in 24% (16/48), and mixed type in 13% (9/68) of heart specimens in this study. The composition of TV studied by authors based either on observation or on histological grounds was membranous [5,7,17].

The assessment of the extent of coverage of the CSO by the TV revealed that in 63 hearts, the occlusion was <75%. In five hearts (7%), the ostial coverage was found to be >75%. Out of these five hearts, two cases in fold type and one case in fenestrated type were completely closing the CSO, which constituted 4% (3/68) and were designated as obstructive TV, potentially complicating CS cannulation. The definition of obstructive TV shown in Table 5 demonstrates that authors mainly focused on a percentage determination of the degree of coverage of the CS by the valve. In most studies, this limit is established to be >75% coverage. According to Holda et al., the obstructive TV was type III (fold type) covering 100% of the CSO. In this study, three heart specimens showed the TV completely closing the CSO.

**Table 5:** Obstructive TV in various studies. *: Cadaveric hearts were formalin-fixed. TV: Thebesian valve; CS: coronary sinus; CSO: coronary sinus orifice; MDCT: multidetector computed tomography

Previous studies	Sample size	Sample type	Occurrence of TV	Definition of obstructive TV	TV might potentially complicate CS cannulation
The present study	104	Fresh autopsied hearts	68 (65%)	TV covering 100% of the CSO	3 (4%)
Kulkarni et al., 2019 [17]	50	*Cadaveric	45 (90%)	TV covering >50% of the CSO	8 (16%)
Holda et al., 2015 [6]	273	*Cadaveric	224 (82.1%)	Type III fold-type TV covering 100% of the CSO	7 (3%)
Ghosh et al., 2013 [7]	150	*Cadaveric	118 (79%)	Non-membranous, non-fenestrated TV covering >75% of the CSO	27 (18%)
Randhawa et al., 2013 [8]	50	*Cadaveric	32 (64%)	TV covering >75% of the CSO	8 (16%)
Katti and Patil 2012 [16]	50	*Cadaveric	44 (80%)	TV covering >65% of the CSO	10 (20%)
Mak et al., 2009 [5]	75	*Cadaveric	55 (73%)	Non-fenestrated, fibrous, fibromuscular, or muscular TV covering >75% of the CSO	12 (16%)
Anh et al., 2008 [10]	98	In vivo	53 (54%)	TV covering >70% of the CSO	11 (11%)
Christiaens et al., 2008 [11]	50	MDCT	18 (36%)	TV covering >60% of the CSO	1 (2%)
Hellerstein and Orbison, 1951 [9]	150	*Cadaveric	128 (85%)	CSO completely covered by the TV	37 (24.7)

Several studies demonstrated the usefulness of both non-invasive and invasive visualization methods which can identify the morphology of the valve before invasive procedures. Scholten et al. in a case report demonstrated the usefulness of intracardiac echocardiography to identify the anatomical variations of TV which helped in the successful catheterization of CS [18]. Shinbane et al. in a case report described how the identification of prominent TV by electron-beam computed tomography angiography helped to choose the epicardial approach rather than the endocardial approach to perform left ventricular lead placement [19]. Kuroda et al. used intraoperative transesophageal echocardiography to successfully guide the cannulation of CS despite the presence of large dynamically moving TV obstructing the CSO during diastole [20]. Mlynarski et al. emphasized the usefulness of cardiac magnetic resonance in the evaluation of CS and its valve before electrophysiological procedures [21]. Thus, from the above studies, it is understood that pre-procedural imaging helps surgeons, electrophysiologists, and cardiac interventionists in the decision-making process and choosing the most optimal equipment and approach to successfully perform cardiac procedures.

## Conclusions

The cannulation of CS is required to perform various diagnostic and therapeutic cardiac procedures which can become difficult or unsuccessful due to the presence of large, fibrous, dynamic, and anomalously originating TV. Thus, pre-procedural imaging of CS and its valve should be integral while planning for CS cannulation. This study highlights the variability in the morphological structure of the TV and its potential implications in unsuccessful CS cannulation and failure of invasive cardiac procedures. Thus, detailed anatomical knowledge about the morphology of the TV is pivotal to planning and adapting procedural strategies during various invasive cardiac procedures.
